# Comorbid Conditions in Temporomandibular Disorders Myalgia and Myofascial Pain Compared to Fibromyalgia

**DOI:** 10.3390/jcm10143138

**Published:** 2021-07-16

**Authors:** Golnaz Barjandi, Eva Kosek, Britt Hedenberg-Magnusson, Ana Miriam Velly, Malin Ernberg

**Affiliations:** 1 Scandinavian Center for Orofacial Neuroscience (SCON), Department of Dental Medicine, Karolinska Institute, SE141 04 Huddinge, Sweden; britt.hedenbergmagnusson@ki.se (B.H.-M.); Malin.Ernberg@ki.se (M.E.); 2 Department of Clinical Neuroscience, Karolinska Institute, SE17 165 Stockholm, Sweden; Eva.Kosek@ki.se; 3 Department of Surgical Sciences, Uppsala University, SE751 85 Uppsala, Sweden; 4 Department of Orofacial Pain and Jaw Function, Eastman Institute, SE113 24 Stockholm, Sweden; 5 Faculty of Dentistry, McGill University, Montreal, QC H3T 1E2, Canada; ana.velly@mcgill.ca; 6 Department of Dentistry, Jewish General Hospital, Montreal, QC H3T 1E2, Canada; 7 Lady Davis Institute for Medical Research, Jewish General Hospital, Montreal, QC H3T 1E2, Canada

**Keywords:** chronic pain, musculoskeletal pain, comorbidity, self-reports, depression, anxiety, irritable bowel syndrome, insomnia, psychological distress, catastrophization

## Abstract

The impact of comorbidities in fibromyalgia (FM) and temporomandibular disorders (TMD) have been well documented, but whether TMD sub-diagnoses myalgia (MYA) and myofascial pain with referral (MFP) differ regarding comorbidity is unclear. We aimed to elucidate this by studying the presence and associations of comorbidities in FM, MFP and MYA. An extended version of the Diagnostic Criteria for TMD axis II questionnaire was used to examine demographics, pain and comorbidities in 81 patients with FM, 80 with MYA, and 81 with MFP. Patients with MFP and FM reported a higher percentage of irritable bowel syndrome (IBS), depression, anxiety, somatic symptoms, perceived stress, and insomnia compared to MYA. Patients with FM had more IBS, depression, and somatic symptom disorder versus MFP. After adjusting for confounding variables, participants with anxiety, somatic symptoms disorder, pain catastrophizing, and perceived stress, as well as a greater number of comorbidities, were more likely to have MFP than MYA, whereas FM participants were more associated with IBS, somatic symptoms and insomnia compared to MFP. The number of comorbidities was significantly associated with widespread pain but not pain duration, body mass index or being on sick leave. In conclusion, patients with MFP were more similar to those with FM regarding comorbidity and should be differentiated from MYA in clinical settings and pain management.

## 1. Introduction

Pain is a highly complex condition, defined as ‘an unpleasant sensory and emotional experience associated with, or resembling that associated with, actual or potential tissue damage’ [1]. It is a multidimensional phenomenon and perhaps more importantly, an individual experience, influenced by memories, cultural background and present life situations [1]. Chronic pain has a predicted prevalence of 20% in Europe and is more common in women compared to men [2]. Musculoskeletal pain encompasses 90% of all chronic pain conditions and is recognised as a major part in the global burden of pain [3]. Musculoskeletal pain can present in different forms, including a wide-spread bodily pain as in fibromyalgia (FM) [4,5], or pain localized to a specific body region like the masticatory muscles in temporomandibular disorders (TMD) [6].

FM is a well-known type of wide-spread chronic musculoskeletal pain with an occurrence of 2 to 4% in the adult population. Patients with FM often complain of increased tenderness in muscles, stress, morning stiffness, imbalance, body mass index (BMI) and fatigue [5,7]. In the orofacial region, TMDs are the most frequent type of chronic pain affecting 5 to 12% of the population. TMD is a collective term referring to pathological conditions in the masticatory muscles and temporomandibular joints [6]. Among these, myogenous TMD is the most common type in clinical settings. Myogenous TMD is characterized by regional or localized pain of the masticatory muscles, but share many of the characteristics of FM, including increased muscle tenderness, pain sensitivity and fatigue [6]. In the validated Diagnostic Criteria for TMD (DC/TMD), myogenous TMD is sub-diagnosed to myalgia (MYA) and myofascial pain with referral (MFP) [6]. This means that if palpation of the masticatory muscle causes pain only at the site of palpation, it is classified as MYA (local pain), and if it refers to structures outside of the muscle, it is classified as MFP (regional pain) [6]. TMD and FM often co-exist in clinical settings and, similar to other chronic pains, they are more prevalent in women [8]. Both conditions often also report high comorbidity with insomnia, irritable bowel syndrome (IBS), somatic symptoms and increased psychological distress (e.g., depression, anxiety, pain catastrophizing) [8,9,10,11].

It is suggested that as pain becomes chronic, the area of pain appears to broaden and involve other structures, leading to an enhanced pain perception and development of comorbid conditions [12]. There is sufficient evidence supporting the relationships between widespread pain and comorbid conditions in patients with FM and TMD [8,9,11,13,14,15]. For instance, the presence of multiple comorbidities not only increases the risk of developing FM and TMD but has also been shown to contribute to persistence of pain and treatment failure [14,16,17]. Although evidence and research regarding comorbidities within both conditions is mounting and the diagnostic tools for both FM and TMD have been refined (to include parameters beyond pain) [5,6], the diagnosis and management of the ‘comorbid patient’ frequently presents as a challenge to healthcare providers. As a result, accurate diagnosis and treatment of these patients may take several years, accentuating the individuals suffering as a whole. Chronic musculoskeletal pain is consequently a common reason for sick leave, placing a huge economic burden on society in terms of healthcare services, loss of workforce, and decreased productivity [18]. There is a need to increase our knowledge within this field to improve management strategies and prognosis of these conditions in order to potentially reduce patient suffering and lessen the economic burden.

Although well-studied in FM and other TMDs [8,9,10,11,15,17,19,20,21], the presence and associations of comorbidities has yet to be explored among patients with MYA and MFP distinguished from each other or in relation to FM. Likewise, it is unclear whether these sub-diagnoses are relevant regarding demographics, pain variables, and BMI. TMD with widespread pain have previously reported a higher presence and stronger associations of comorbidities compared to those without [15,22]. In addition, longitudinal studies have found that the presence of comorbidity and widespread pain in patients with TMD leads to persistence of pain and poor treatment outcomes [23,24,25]. Considering that pain spreading is a part of the MFP diagnosis, exploring the presence and associations of comorbidities in FM, MFP, and MYA is relevant and could highlight possible intervention strategies. Accordingly, the primary aim of this study was to compare the presence of comorbid conditions between women with FM, MFP, and MYA and to investigate their associations with pain diagnoses. Secondly, we aimed to examine the associations between the number of comorbid conditions and pain duration, widespread pain, being on sick leave, and BMI. Based on existing literature, the null hypotheses examined whether there are no significant differences between FM, MYA, and MFP regarding comorbid conditions; and also whether an increasing number of comorbidities is associated with widespread pain, pain duration, being on sick leave, or BMI.

## 2. Materials and Methods

### 2.1. Study Design

This was a cross-sectional study investigating self-reported comorbidity in women diagnosed with FM, MFP, and MYA. This study was approved by the regional ethical review board in Stockholm, Sweden and followed the guidelines according to the declaration of Helsinki. Upon enrolment, all participants had signed an informed consent. The data were collected between the years 2017 and 2020. The participants were recruited among consecutive patients referred to the Department of Dental Medicine at Karolinska Institutet (Huddinge, Sweden), Stockholm Spine Center (Stockholm, Sweden), or by advertisement. All participants completed an extended version of the standardized and validated Swedish version of the DC/TMD axis II questionnaire, including variables regarding demographics (including age, birthplace, education level, marital status, and occupation), BMI, pain duration, and comorbidities.

### 2.2. Participants

Prior to the start of the study, a sample size calculation was performed. Assuming a small difference of 30% and a standard deviation of residuals of 45% as clinically significant for the total number of comorbid conditions between groups, 80 participants in each group would yield a power of more than 90% using a significance level of 5%.

Inclusion criteria were a diagnosis of FM, MYA, or MFS, an age between 18 and 75 years, and female sex. The reason for only including women was to avoid gender differences as a potential confounding factor for the analysis. As noted previously, both FM and TMD patients are predominantly female [8]. Exclusion criteria included male sex but also an inability to understand the Swedish language, falling outside the age range, an incomplete questionnaire, or any condition that interfered with completing the questionnaire

#### 2.2.1. Fibromyalgia

Participants diagnosed with FM were either recruited via advertisement through the Swedish Rheumatism Association and the Fibromyalgia Association of Sweden or among consecutive patients at Stockholm Spine Center, diagnosed using the American College of Rheumatology (ACR) revised 2016 criteria [5].

#### 2.2.2. Temporomandibular Disorders

Participants with TMD were recruited among consecutive patients referred to the Orofacial Pain Clinics at the Department of Dental Medicine at Karolinska Institutet in Huddinge, Sweden. All participants fulfilled a diagnosis of either MYA or MFP according to the DC/TMD [6] and had a pain duration of at least 3 months. Additional exclusion criterion to those described above was a pre-existing diagnosis of FM.

### 2.3. Widespread Pain

The widespread pain index (WPI) is a self-report measure that assesses and quantifies the level of widespread pain in the body [26,27]. The WPI assesses the presence of pain in 19 specific body regions over the past seven days, divided into axial (chest, abdomen, neck, upper back, lower back), upper body right and left side (jaws, shoulders, upper arms, lower arms) and lower body right and left side (hips, upper legs, lower legs). Each item/pain region is equivalent to a score of 1. The total number of regions (0–19) is calculated as WPI, with a higher score indicating more widespread pain. A score 0–5 is considered as local pain, whereas a score of 6–19 is considered as widespread pain [5].

According to the ACR 2016 criteria [5], the WPI in conjunction with the Symptom Severity Scale (SSS) can be used to diagnose FM. The SSS consists of three questions about fatigue, tiredness, and cognitive problems that are answered on a Likert scale ranging from 0 to 3 where 0 is ‘no symptoms’ and 3 corresponds to ‘severe symptoms’. For participants with MFP and MYA, these two scales were used to detect self-reported FM. In order to receive a self-reported FM diagnosis, the participants should report: ‘(1) a WPI score ≥ 7 and SSS score ≥ 5 or a WPI 4 to 6 and SSS ≥ 9; (2) a generalized pain defined as pain in at least 4 out of 5 body regions (axial, upper right, upper left, lower right, lower left; where jaws, chest and abdomen are excluded); and (3) pain symptoms present for at least 3 months’ [5].

### 2.4. Specific Comorbid Conditions

The presence of seven comorbid conditions (IBS, depression, anxiety, somatic symptoms, pain catastrophizing, perceived stress, insomnia) was identified using a battery of validated instruments, presented below. The specific comorbid conditions examined in this study was based on the growing evidence of their involvement in FM and TMD [8,9,10,11] and are included in an extended version of the DC/TMD axis II questionnaire used in the Specialist Clinic for Orofacial Pain at the University Dental Clinic, Karolinska Institutet.

#### 2.4.1. Irritable Bowel Syndrome

Participants were evaluated for comorbid IBS using the Rome IV Criteria [28]. The Rome IV Criteria defines GI disorders as a function of the gut–brain interaction, meaning that many different body processes can lead to a functional GI disorder. The questionnaire consists of five questions, wherein the first assesses the frequency of bowel pain ranging from ‘never’ (0) to ‘several times every day’ (8). The second question assesses the duration of bowel pain, defined as more or less than 6 months. Thereafter, questions three to five evaluate the frequency (in percent) of bowel pain that is associated with defecation as well as variation in the regularity and shape of faeces. In order to receive a diagnosis of IBS using the Rome IV Criteria, the patient needs to present with repeated abdominal pain a minimum of once day per week in the last three months (score 4, question 1) with manifestation of symptoms 6 months prior or more (question two), and report a frequency of 30 percent or higher on at least two out of questions three to five [28].

#### 2.4.2. Depression

Participants were evaluated for symptoms of depression such as tiredness, bad appetite, loss of interest/pleasure experienced during the last two weeks using the 9-item Patient History Questionnaire (PHQ-9) [29]. The PHQ-9 is a reliable and valid instrument which was initially developed as a diagnostic tool. It consists of 9 questions in which the participant can choose from answers on a Likert scale ‘never’ = 0, ‘several days’ = 1, ‘more than half of the days’ = 2, and ‘almost every day’ = 3. The scores range from 0 to 27, where cut-off points 5, 10, 15, and 20 represent mild, moderate, moderate severe, and severe levels of depression, respectively. The PHQ-9 can also be used categorically to detect the presence of a depressive disorder, in which case a cut-off score of 10 or more is accepted as clinically relevant and has a specificity and sensitivity of 88% [29].

#### 2.4.3. Anxiety

Participants were evaluated for anxiety symptoms such as feelings of nervousness, irritation, and restlessness during the last 2 weeks using the reliable and validated Generalized Anxiety Disorder-7 (GAD-7). It consists of 7 questions in which the participant can chose from answers on a Likert scale ‘never’ = 0, ‘several days’ = 1, ‘more than half of the days’ = 2, and ‘almost every day’ = 3. The scoring of GAD-7 ranges from 0 to 21, where cut-off points of 5, 10, and 15 represent mild, moderate, and severe levels of anxiety, respectively. A score of 10 or more indicates the presence of an anxiety disorder with a sensitivity of 89% and a specificity of 82% [30].

#### 2.4.4. Somatic Symptoms

Presence of somatic symptoms was measured in participants using the 15-item PHQ. This questionnaire includes the most prevalent symptoms included in the DSM-IV somatization disorder (renamed to somatic symptom disorder in DSM-V) [31]. Participants were asked to rate the severity of symptoms such as bowel pain, headache, dizziness, and fainting spells during the last 4 weeks on a Likert scale where 0 = ‘not bothered at all’, 1 = ‘bothered a little’, and 2 = ‘bothered a lot’. The scoring ranges from 0 to 30, where cut-off points of 5, 10, and 15 represent mild, moderate, and severe levels of somatic symptoms, respectively. A score of 10 or more indicates the presence of a somatic symptom disorder. Previous studies have found that the use of PHQ-15 has a high reliability and validity in healthcare [32,33].

#### 2.4.5. Pain Catastrophizing

The pain catastrophizing scale (PCS) was used to help quantify participants’ individual pain experience, asking about how they feel and what they think about when they are in pain [34]. It consists of 13 questions where participants are asked to indicate the degree to which they have the abovementioned thoughts/feelings when experiencing pain using a Likert scale of 0 (not at all) to 4 (all the time). The scoring of PCS ranges from 0 to 52, where cut-off points of 20 and 30 represent the risk and the high risk of pain catastrophizing, respectively. A score of more than 30 or more is the validated cut-off for clinically relevant pain catastrophizing [34].

#### 2.4.6. Perceived Stress

The Perceived Stress Scale (PSS-10) was used to evaluate how often the participants had experienced life as unpredictable, uncontrollable, or overburdening. PSS-10 is a widely used questionnaire with a high reliability and validity [35]. It consists of 10 questions where the participant answers using a Likert scale of 0 = ‘never’ to 4 = ‘very often’. The scoring ranges from 0 to 40, where cut-off points of 13 and 21 represent the moderate and high level of perceived stress, respectively. There are no official cut-off points when interpreting PSS-10. A previous study on psychometric evaluation and normative data of the Swedish version of PSS-10 reported the average score of 13.5 [35], and hence a score of 15 or over could indicate stress problems. Based on this, the cut-off score for comorbid perceived stress was put at 15 in our study.

#### 2.4.7. Insomnia

The Insomnia Severity Index (ISI) was used to evaluate sleep disturbances in participants. It consists of seven questions evaluating sleep difficulties, early wakeups, being well-rested, and the effects of participant sleeplessness (among others). The participants used answers on a Likert scale from 0 = ‘never’ to 4 = ‘very often’. The scoring of ISI ranges from 0 to 28, where cut-off points of 8, 15, and 22 represent mild, moderate, and severe insomnia, respectively. A score of than 15 or more represents clinically significant insomnia [36].

### 2.5. Number of Comorbid Conditions

The total number of comorbid conditions in this study is based on the abovementioned seven questionnaires (i.e., Rome IV Criteria for IBS, PHQ-9, GAD-7, PHQ-15, PCS, PSS-10, and ISI). The presence of specific comorbid conditions (IBS, depression, anxiety, somatic symptoms disorder, pain catastrophizing, perceived stress, insomnia) was identified using validated and clinically relevant cut-off points (stated above). From these, the total number of comorbid conditions for each participant was calculated as the sum of all the above-mentioned comorbid conditions. Hence, a participant could have a maximum of seven comorbid conditions.

### 2.6. Statistical Analyses

The statistical analyses were performed using SigmaPlot v.14.0 (Systat Software, Inc., San Jose, CA, USA) and SPSS 27.0 (IBM Corp, Armonk, NY, USA). A threshold of alpha level equal to 0.05 was used for statistical significance. Pain diagnosis (i.e., MYA, MFP or FM) was considered the dependent variable whereas demographics, BMI, pain duration, widespread pain, and comorbidities (i.e., IBS, depression, anxiety, somatic symptoms disorder, pain catastrophizing, perceived stress, insomnia) were defined as independent variables. Descriptive statistics were used to characterize the sample and compare demographics, pain variables, and comorbidities between participants with MYA, MFP, and FM. As the variables analyzed in the present study were either ordinal or had a non-normal distribution, non-parametric statistics (chi-squared test or ANOVA on ranks/Kruskal–Wallis test) were used to identify the statistical differences between the pain groups. The Bonferroni correction test and Dunn’s test were used to adjust for multiple comparisons in case of significant differences. We used a multinomial logistic regression model analysis to test for the associations between the pain groups and (1) each specific comorbid condition as well as (2) the number of comorbid conditions. When setting up our analysis, individuals with MFP were set as a reference group against which MYA and FM were compared. To further explore whether the number of comorbidities was associated with widespread pain, pain duration, sick leave, and BMI, an ordinal logistic regression was performed. In this model, the number of comorbidities was considered as the dependent variable. Multiple imputation with chained equations was used for missing data. All the above-mentioned regression models were controlled for demographics, BMI, pain duration, and widespread pain.

## 3. Results

### 3.1. Sample Characteristics

Out of 434 participants that were offered to participate in the study, 123 participants dropped out before enrolment. Upon enrolment, 69 participants (FM: 24, TMD: 45) were excluded for not completing the questionnaires used to detect comorbidities, voluntary drop-out, or due to a previous diagnosis of FM (for TMD participants). As a result, a total of 242 participants were included in the study (80 MYA, 81 MFP, and 81 FM). Table 1 summarizes participants’ demographics, BMI, pain variables, and comorbidity scoring by pain diagnosis (i.e., MYA, MFP, FM).

#### 3.1.1. Demographics

Overall, the median participant age was 50.1 (IQR: 18.2) years and did not differ significantly between the groups. Although most participants (77.3%) were born in Scandinavia, the FM group differed significantly from the MYA and MFP groups. Moreover, significantly more participants with FM were married or were co-habitants compared to MFP, but not compared to MYA. Almost 50% of participants with FM and 30% with MFP were reported to be on sick leave, which differed significantly to the MYA group (5.1%). A significant difference was also found between the FM and MFP groups regarding sick leave. No significant difference could be found between the pain groups regarding educational level (Table 1).

#### 3.1.2. BMI

Participants with FM had a significantly higher median self-reported BMI compared to both MFP and MYA (Table 1).

#### 3.1.3. Pain Duration and Widespread Pain

Median and interquartile range (IQR) pain duration and WPI score can be found in Table 1. Participants with FM reported a median pain duration of 20.5 (IQR 20.0) years, which was significantly longer compared to both MYA and MFP. Participants with MFP also reported a significantly longer median pain duration compared to MYA. The median WPI differed significantly between all pain groups, where FM reported the highest median WPI score and MYA reported the lowest. Only WPI scores for FM and MFP indicated a presence of widespread pain (WPI > 5).

### 3.2. Specific Comorbid Conditions

Table 1 shows the scoring of the questionnaires included in the study to assess comorbidity. Participants with MYA scored significantly lower on all scales compared to the other pain groups, except for pain catastrophizing, where MYA differed significantly only from those with MFP. FM and MFP differed significantly regarding the level of somatic symptoms, where MFP reported a lower median score.

The presence of specific comorbid conditions in the study sample can be found in Table 2. Significantly more participants with MFP fulfilled the ACR 2016 criteria for FM compared to MYA (45.7% versus 12.5%, *p* < 0.001). Because of this high comorbidity, we compared MFP participants with and without FM regarding demographic, pain, and comorbidity variables and found only significant differences regarding the presence of somatic symptoms and mean (SD) BMI (See Supplementary Materials, Table S1).

Compared to MYA, participants with MFP and FM reported a higher percentage of self-reported IBS, depression, anxiety, somatic symptoms disorder, perceived stress, and insomnia (Table 2).

Participants with FM had a significantly higher percentage of self-reported IBS, depression, and somatic symptoms disorders compared to MFP. On the other hand, MFP had a significantly higher percentage of pain catastrophizing compared to MYA and FM participants, but the difference was only significant when compared to MYA (Table 2).

The associations between each comorbid condition and pain diagnosis are shown in Table 3. After adjusting for demographics, BMI, and pain duration, we found that MYA participants were significantly less associated with comorbid depression, anxiety, somatic symptoms disorder, pain catastrophizing, perceived stress, and insomnia as compared to participants with MFP. FM participants were significantly more likely to present with comorbid IBS, depression, and somatic symptoms disorder, both in the crude and in the adjusted model.

### 3.3. Number of Comorbid Conditions

Participants with FM and MFP reported a significantly higher number of total comorbidities compared to MYA (FM = 5.0 (IQR 3.0), MFP = 4.0 (IQR 3.5) versus MYA = 1.0 (IQR 3.8), *p* < 0.001). Participants with MFP and FM did not differ significantly from each other regarding the number of comorbidities. The associations between the number of comorbid conditions and pain diagnosis are shown in Table 4. The odds ratio of 0.69 for MYA versus MFP (*p* = 0.006) indicate that participants with a higher number of comorbid conditions were more likely to be MFP participants than those with a lower number of comorbid conditions. No significant differences were found when FM was compared to MFP.

Table 5 shows a summary of the generalized ordinal logistic regression analysis of the association between the number of comorbid conditions and widespread pain, pain duration, being on sick leave, and BMI. There was a significant positive association between increased widespread pain and a greater number of comorbidities. Pain duration and being on sick leave were also positively associated with a greater number of comorbidities. The total number of comorbid conditions was not related to BMI. All of the above-presented associations were controlled for age, demographics, BMI, and pain duration but not for widespread pain. However, when widespread pain was included in the multivariable model, the association between pain duration or being on sick leave and the number of comorbidities were no longer significant.

## 4. Discussion

This cross-sectional study showed that participants with self-reported anxiety, somatic symptoms disorder, pain catastrophizing, and perceived stress, as well as a greater total number of comorbidities, were significantly more likely to have a TMD sub-diagnosis of MFP compared to MYA. On the other hand, FM in comparison to MFP only reported a significant association with IBS, somatic symptoms disorder, and insomnia. We also found that widespread pain, pain duration, and being on sick leave, but not BMI, increased the likelihood of having more comorbidities. Interestingly, when including widespread pain in the multivariable model, a significant association was only found between widespread pain and number of comorbidities, suggesting that the pain duration and sick leave were related to the likelihood of increasing comorbidities and may be modified by widespread pain.

This is the first study, to the best of our knowledge, that explores the relationships between comorbidities in women with MYA, MFP, and FM. The results of this study corroborated previous data regarding the strong association between comorbidities and widespread pain [8,9,10,11,15,17,19,20,21], while also revealing some novel findings such as the clear differences between the sub-groups of TMD and their different likelihoods of reporting comorbidities, where those with MFP presented with a profile more similar to the FM group.

The percentage of most self-reported comorbidities, as well as the number of comorbid conditions in this study, were the highest for FM and the lowest for MYA, whereas MFP mostly represented an intermediate group between MYA and FM. Notably, MFP and FM did not differ significantly regarding the number of comorbidities they presented with. Self-reported somatic symptoms disorder and depression differed the most between MYA, MFP, and FM, which coincides with similar previous research in patients with and without widespread pain [15,22,37,38]. For instance, previous findings show that 88.9% with FM and 28.5–76.6% with TMD (unknown sub-diagnosis) presented with moderate to severe somatic symptoms [39,40]. Somatic symptoms are common among FM patients and are also closely related to widespread pain and disability [41], which could explain its high comorbidity with FM found in our study, as well as in other studies. Overall, the observed presence of comorbidities increasing from MYA via MFP to FM agrees with previous similar studies [42,43,44,45]. In our study, widespread pain differed significantly between the groups and was the highest in FM and the lowest in MYA, which was expected as the diagnosis of both FM and MFP require the spread of pain [5,6]. Widespread pain has previously also been found to correlate linearly with pain duration, as well as negatively impacting biopsychosocial factors, work-status, and patients’ quality of life [46,47,48]. This may partly explain the differences found in our study regarding pain duration and sick leave between all the pain groups. Moreover, the substantial difference in the frequency of MFP patients being on sick leave compared to MYA (30 versus 5 percent) is notable and reflective of the increased pain-related disability and poor treatment outcomes previously found in TMD patients with pain spreading [49,50]. These results indicate that MFP could be a more severe condition compared to MYA and perhaps a transition phase towards FM [51,52], highlighting the importance of the early detection/intervention of these patients (e.g., by screening, education, and raising awareness) as well as differentiation between MFP and MYA in clinical settings.

Our results demonstrate stronger associations of a specific and greater number of comorbidities in MFP compared to MYA, and were overall in line with previous similar/relevant research demonstrating sub-group differences between those with localized and widespread/generalized pain [14,15,22,53]. For instance, patients with pain in multiple pain regions have reported relevant associations with anxiety [54], psychological distress [55], insomnia, and somatic symptoms [15,22] as well as multiple comorbid conditions compared to those without widespread pain. In contrast to our results, studies of TMD patients with widespread pain have previously found additional significant associations with, e.g., IBS [22]. Probable explanations for these discrepancies could be due to differences in classification of pain spreading and the analysis methods used. Regardless, although causality cannot be evaluated due to the design of the present study, these findings raise the hypothesis that MYA and MFP could have separate mechanisms that may contribute to their distinct pain and comorbidity characteristics. Previously, the presence of comorbidities in TMD has signified a more complex disorder and been explained as a dysregulation of pain modulatory systems involving central and peripheral sensitization [56]. This could additionally explain our findings of increased pain duration and widespread pain in MFP (compared to MYA) and that it may be a transition phase to FM. Interestingly, Jasim et al. (2020) [57] recently reported that patients with MYA and MFP differed from each other in the salivary expression of some metabolic, stress, and immune-related proteins, which supports our hypothesis and additionally suggests a potential role for these proteins.

On the other hand, we found that FM was only significantly associated with self-reported IBS, depression, and somatic symptoms. Previous observational studies have found that TMD and FM, compared to their pain-free counterparts, are more likely to have depression, IBS, and somatic symptoms, as well as many other comorbidities [14,58,59]. The lack of significance regarding other associations between FM and MFP is unclear but could be due to the high presence of self-reported FM in the participants with MFS (45.6%) (although TMD participants with a pre-diagnosed FM were excluded). Previous studies have shown that the prevalence of FM in TMD patients varies from approximately 7% to 60% [60], depending on e.g., study methods and/or TMD diagnosis used. Nevertheless, having a widespread pain, such as FM, has previously been linked to altered pain processing, dysregulation of the immune system, and brain alterations [60]. It has also been found to negatively impact biopsychosocial conditions of patients [60]. For instance, TMD patients with FM have reported more severe and sustained pain, more comorbidities, worse TMD problems and treatment difficulties compared to those without [61]. The high comorbidity and similarities in patient characteristics between FM and MFP found in this study may suggest that these two conditions could share common pathophysiological pathways.

We also found that increased widespread pain, pain duration, or being on sick leave increased the likelihood of presenting more comorbidities, which are in line with previous research in both FM and TMD, but also other chronic pain conditions. However, the association between comorbidities, pain duration, and sick leave did not remain significant in the multivariable model when we adjusted for widespread pain, suggesting that widespread pain might play a role in these associations. Similarly, exploratory analysis showed that the associations of depression, IBS, insomnia, and somatic symptoms between the pain diagnosis were modified by WPI (see Supplementary Materials, Table S2), further indicating a role of widespread pain in these associations. The reason for these findings is unclear. Although controversial, it has previously been proposed that overlapping comorbidities such as widespread pain, FM, some cases of TMD, IBS, sleep disturbances, depression, and somatic symptoms should be considered as a single entity (such as ‘Overlapping Chronic Pain Conditions’) rather than many distinct conditions with separate etiologies [62]. This is because these conditions often share similar patient characteristics/symptoms, treatment difficulties, and pathophysiology. Thus, the similarities between MFP and FM and the potential modifying effect of widespread pain in this study could be due to these patients being a part of this continuum of overlapping comorbidities. Future studies are warranted to elucidate this hypothesis.

There are some limitations to our study. Firstly, a major limitation is that approximately 50 percent of the participants with MFP fulfilled the criteria for FM according to ACR 2016, although MFP patients with a pre-existing FM were excluded. In addition to this, we did not control for TMD in the FM group. As a result, the comparisons in this study may be biased and ambiguous. Due to this, we compared MFP participants with and without FM regarding demographic, pain, and comorbidity variables and found only significant differences regarding the presence of somatic symptoms disorder and BMI (See Supplementary Materials, Table S1). Secondly, we used self-reported questionnaires to detect comorbidities and widespread pain, which may have given rise to recall bias. Although the questionnaires (and cut-off points) used in this study were validated, there is the possibility of nondifferential misclassification due to patient overreporting that in turn may have exaggerated the associations found in this study. Thirdly, patient history regarding pre-diagnosed comorbidities, medications, or current undergoing treatment was not taken into consideration and may have impacted on our results. Our findings may therefore be biased as a result of unmeasured confounding variables not included in the study. Fourth, due to our study design, the direction of associations found in the present study are ambiguous. Fifth, we only included female pain participants in the current study, which as a result limits the generalizability of our findings. Although both TMD and FM have a female predominance [8], future studies should include males to study potential sex differences. Lastly, we have to discuss the possibility of selection bias in our study. Selective recruitment may have occurred because of the relatively long and demanding questionnaire that potentially could lead to some participants not participating in the study or not completing the questionnaire. Moreover, pain patients were recruited from tertiary care clinics and our findings may therefore not be generalizable to the general population. Future studies should address these limitations.

The main strength of this study is that we classified and differentiated between widespread, regional, and local pain using specific pain diagnoses based on valid, internationally accepted criteria; the DC/TMD and ACR 2016 [5,6]. Using MFP as a reference in our analysis allowed us to easily detect how this pain group differentiates from MYA and FM. Possible confounder variables such as age, country of birth, education, and work-status were collected for adjustment purposes. In addition, we excluded pre-diagnosed FM to improve the precision of the TMD classification and comorbid status. Based on our power calculation, the sample size included in the current study can establish clinically significant associations of comorbidities in these pain groups. As a result, this study has several clinical implications. Our findings indicate MFP to be a more complex pain condition compared to MYA, and perhaps a transition phase to FM as they retained clinical, pain, and comorbidity characteristics similar to FM that previously also have been found to complicate TMD pain management and worsen prognosis [56,61]. Hence, this study highlights the importance of differentiating between MFP and MYA and further confirms the need to acknowledge the presence of comorbidities and widespread pain in the evaluation, management, and intervention strategies. Failure to recognize and manage the entire pain problem, co-morbidities, and other contributing factors may lead to treatment failure and further prolong the pain [16,56]. Evaluation of these pain groups, using questionnaires or comparable patient interviews, can help identify comorbidities and widespread pain in an easy way and thus potentially improve pain management. If identified, these patient cases could be acknowledged as more complex and may best be managed using an interdisciplinary approach with relevant medical specialties working together [56].

## 5. Conclusions

Overall, our results showed that the presence of comorbidities were significantly more associated with MFP compared to MYA, whereas FM and MFP did not differ as much from each other. Given the similarities between MFP and FM, these findings not only highlight the importance of differentiating between MFP and MYA in clinical settings, but also raise the hypothesis that MFP may be a transition phase towards FM. Moreover, this study also confirms the need for recognizing widespread pain and comorbidities in assessments of these pain groups and that the presence of one should raise suspicion of the other. As management of comorbidities in chronic pain conditions result in greater success in long-term treatment and prognosis [56], it is important to detect these patients early and provide a multimodal treatment approach. Further studies are warranted for evaluating and identifying the biological mechanisms underlying these associations and potential multimodal intervention strategies for improving pain management.

## Figures and Tables

**Table 1:** Descriptive characteristics of 80 women with myalgia (MYA), 81 with myofascial pain (MFP), and 81 with fibromyalgia (FM).

	MYA	MFP	FM
Age, years, median (IQR)	49.4 (25.7)	45.7 (19.9)	53.3 (14.5)
Born in Scandinavia, *n* (%)	63 (78.9)	60 (74.1)	75 (92.6) ^a,b^
University graduate, *n* (%)	40 (50.0)	39 (48.1)	39 (48.1)
On Sick leave, *n* (%)	5 (6.2)	25 (31.0) ^A^	39 (48.1) ^A,b^
Married/Co-habitant, *n* (%)	50 (61.7)	45 (55.6)	58 (71.6) ^b^
BMI, median (IQR)	24.0 (4.6)	24.3 (7.7)	28.4 (7.6) ^A,B^
Pain duration, median, yr (IQR)	2.0 (4.1)	5.0 (8.0) ^a^	20.5 (20.0) ^A,B^
WPI, median (IQR)	4.0 (4.0)	8.0 (7.5) ^A^	14.0 (7.0) ^A,B^
PHQ-9, median (IQR)	5.0 (8.0)	11.0 (10.0) ^A^	13.0 (7.5) ^A^
GAD-7, median (IQR)	3.0 (7.0)	7.0 (11.0) ^A^	7.0 (9.0) ^A^
PHQ-15, median (IQR)	8.0 (7.0)	14.0 (7.5) ^A^	17.0 (5.5) ^A^
PCS, median (IQR)	9.0 (17.6)	21.5 (24.6) ^a^	16.0 (18.0)
PSS-10, median (IQR)	12.0 (13)	20.0 (12.0) ^A^	22.0 (13.5) ^A^
ISI, median (IQR)	10.0 (11.6)	15.0 (11.0) ^a^	17.0 (9.0) ^A^

BMI: Body mass index; PHQ: patient health questionnaire; GAD-7: Generalised anxiety disorder, 7-item scale; PHQ-15: Patient health questionnaire, 15 item scale; PCS: Pain catastrophizing scale; PSS-10: perceived stress scale, 10 items; ISI: Insomnia severity index; IQR: interquartile range; Significance indicated with a capital letter if *p* < 0.001 or with a lowercase letter if *p* < 0.05. ^A or a^ = significant compared to myalgia, ^B or b^ significant compared to myofascial pain with referral.

**Table 2:** Presence of self-reported comorbid conditions in each study group based on questionnaires. Data are presented as number (%) of participants with the specific comorbid condition.

	MYA N (%)	MFP N (%)	FM N (%)
IBS	20 (25.0)	37 (45.8) ^a^	53 (65.4) ^A, b^
Depression	22 (27.5)	45 (55.6) ^A^	63 (77.8) ^A, b^
Anxiety	9 (11.3)	34 (42.0) ^A^	31 (38.3) ^A^
Somatic symptoms	35 (43.8)	63 (77.8) ^A^	75 (92.6) ^A, b^
Pain catastrophizing	10 (12.5)	23 (28.8) ^a^	14 (17.3)
Perceived stress	31 (38.8)	56 (69.1) ^A^	60 (74.1) ^A^
Insomnia	25 (31.3)	56 (69.1) ^A^	55 (67.9) ^A^

MYA: Myalgia; MFP: myofascial pain with referral; FM: fibromyalgia; IBS: Irritable bowel syndrome; Significance is indicated with a capital letter if *p* < 0.001 or with a lowercase letter if *p* < 0.05. ^A or a^ = significant compared to myalgia, ^B or b^ significant compared to myofascial pain with referral.

**Table 3:** Multinomial logistic regression analyses assessing the association between each specific comorbidity and the pain groups (dependent variable). In these analyses, individuals with MFP were set as a reference against which the other groups were compared.

Comorbidity	MYA vs. MFP				FM vs. MFP			
	Crude		Adjusted ^1^		Crude		Adjusted ^1^	
	OR	95% CI	OR	95% CI	OR	95% CI	OR	95% CI
IBS	0.42	0.21–0.81 **	0.53	0.26–1.08	2.37	1.26–4.46 **	2.79	1.26–6.19 **
Depression	0.30	0.16–0.59 ***	0.42	0.20–0.87 *	2.44	1.25–4.76 **	2.69	1.12–6.43 *
Anxiety	0.18	0.08–0.40 ***	0.18	0.07–0.43 ***	0.86	0.46–1.61	1.10	0.49–2.48
Somatic symptoms	0.22	0.11–0.44 ***	0.25	0.12–0.53 ***	3.57	1.34–9.54 **	4.62	1.32–16.2 *
Pain catastrophizing	0.30	0.13–0.70 **	0.34	0.14–0.87 *	0.50	0.24–1.05	0.79	0.29–2.13
Stress	0.27	0.14–0.51 ***	0.30	0.14–0.59 ***	1.20	0.60–2.40	1.21	0.53–2.80
Insomnia	0.33	0.17–0.63 ***	0.47	0.23–0.96 *	1.45	0.76–2.74	1.25	0.56–2.80

MYA: Myalgia; MFP: myofascial pain with referral; FM: fibromyalgia; OR: Odds ratio; CI: confidence interval; IBS: Irritable bowel syndrome; ^1^ Adjusted for age, demographics, BMI, pain duration; Statistical significance is indicated by * *p* ≤ 0.05, ** *p* ≤ 0.01, *** *p* ≤ 0.001.

**Table 4:** Multinomial logistic regression analyses assessing the association between the number of comorbid conditions and diagnosis (dependent variable). In this analysis, individuals with MFP were set as a reference against which the other groups were compared.

	Crude		Adjusted ^1^	
	OR	95% CI	OR	95% CI
MYA vs. MFS	0.64	0.54–0.75 ***	0.69	0.57–0.82 ***
FM vs. MFS	1.14	0.97–1.33	1.23	0.99–1.51

MYA: Myalgia; MFP: myofascial pain with referral; FM: fibromyalgia. OR: Odds ratio, CI: confidence interval. ^1^ Adjusted for age, demographics, BMI, pain duration. Statistical significance is indicated by *** *p* ≤0.001.

**Table 5:** Generalized ordinal logistic regression assessing the association between the total number of comorbid conditions (dependent variable) and WPI, pain duration, sick leave, and BMI in pain groups.

	Crude			Adjusted ^1^			Fully Adjusted Model ^2^		
	B	SE	OR (95% CI)	B	SE	OR (95% CI)	B	SE	OR (95% CI)
BMI	0.04	0.02	1.04 (1.00–1.08)	0.03	0.02	1.03 (0.99–1.08)	0.02	0.03	1.02 (0.97–1.07)
Pain duration	0.04	0.01	1.04 (1.02–1.06) ***	0.03	0.01	1.04 (1.01–1.06) **	−0.00	0.01	1.00 (0.97–1.02)
WPI	0.20	0.02	1.22 (1.16–1.27) ***	0.19	0.04	1.21 (1.13–1.30) ***	0.19	0.04	1.21 (1.13–1.30) ***
Being on sick leave	1.21	0.26	3.34 (2.02–5.55) ***	1.09	0.32	2.98 (1.60–5.54) ***	0.47	0.33	1.60 (0.83–3.08)

WPI: widespread pain index; BMI; body mass index; SE: standard error; OR: odds ratio; CI: confidence intervals. ^1^ Adjusted for age, demographics, body mass index, pain duration. ^2^ Adjusted for age, demographics, body mass index, pain duration, and widespread pain index. Significance is indicated by ** *p* < 0.01, *** *p* < 0.001.

## Data Availability

The raw data supporting the conclusions of this article will be made available by the authors on request, without undue reservation.

## Supplementary Materials

The following are available online at https://www.mdpi.com/article/10.3390/jcm10143138/s1. Table S1: Patient characteristics of 37 MFP participants with FM and 44 MFP participants without FM, Table S2: Fully adjusted/exploratory multinomial logistic regression analyses assessing the association between each specific comorbidity and the pain groups (dependent variable). In this analysis, individuals with MFP were set as a reference against which the other groups were compared.
