# Supplementary material for: Comorbid Conditions in Temporomandibular Disorders Myalgia and Myofascial Pain Compared to Fibromyalgia
Source: J Clin Med. 2021 Jul 16;10(14):3138. doi: 10.3390/jcm10143138 (PMC8306531; doi:10.3390/jcm10143138)
Supplement: Supplementary file 1 [file jcm-10-03138-s001.zip › jcm-1246861-supplementary.pdf]

Table S1. Patient characteristics of 44 MFP participants without FM and 37 MFP participants with FM.

	**MFP without FM**	**MFP with FM**	***p***
Age, years, median (IQR)	44.9 (12.6)	50.0 (12.0)	0.07
BMI, median (IQR)	24.2 (4.1)	27.1 (5.9)	0.02*
Pain duration, median, yr (IQR)	4.0 (7.4)	7.0 (13.0)	0.13
Born in Scandinavia, n (%)	31.0 (77.5)	24.0 (68.6)	0.54
University graduate, n (%)	21.0 (47.7)	14.0 (42.4)	0.53
On Sick leave, n (%)	10.0 (23.3)	14.0 (38.9)	0.21
Married/Co-habitant, n (%)	25.0 (56.1)	19.0 (52.8)	0.95
IBS, n (%)	17.0 (38.6)	19.0 (51.4)	0.36
Depression, n (%)	26.0 (59.1)	19.0 (51.4)	0.64
Anxiety, n (%)	23.0 (52.3)	11.0 (29.7)	0.07
Somatic Symptoms, n (%)	28.0 (63.6)	35.0 (94.6)	0.002**
Perceived Stress, n (%)	29.0 (65.9)	28.0 (75.7)	0.48
Pain Catastrophizing, n (%)	14.0 (31.8)	10.0 (27.0)	0.82
Insomnia, n (%)	22.0 (50.0)	25.0 (67.6)	0.17

MFP: myofascial pain with referral; FM: fibromyalgia; BMI: Body mass index; IBS: Irritable bowel syndrome; Statistical significance is indicated by * = p≤0.05, ** = p≤0.01

Table S2. Fully adjusted/exploratory multinomial logistic regression analyses assessing the association between each specific comorbidity and the pain groups (dependent variable). In this analysis, individuals with MFP were set as reference against which the other groups were compared.

**Comorbidity**	**MYA vs MFP**		**FM vs MFP**	
	OR	95% CI	OR	95%CI
**IBS**	0.79	0.33-1.61	1.46	0.57-3.69
**Depression**	0.47	0.21-1.05	1.53	0.58-4.04
**Anxiety**	0.21	0.08-0.54***	0.70	0.27-1.82
**Somatic symptoms**	0.45	0.20-1.03*	1.18	0.29-4.85
**Pain catastrophizing**	0.36	0.13-0.97*	0.63	0.20-2.02
**Stress**	0.42	0.19-0.91*	0.63	0.24-1.65
**Insomnia**	0.65	0.30-1.40	0.71	0.27-1.84

MYA: Myalgia; MFP: myofascial pain with referral; FM: fibromyalgia; OR: Odds ratio; CI: confidence interval; IBS: Irritable bowel syndrome; Adjusted for age, demographics, body mass index, pain duration, widespread pain index; Statistical significance is indicated by * = p≤0.05, ** = p≤0.01, *** = p≤0.001.
